# The impact of fatigue on people with multiple sclerosis in Morocco

**DOI:** 10.4102/ajod.v13i0.1376

**Published:** 2024-07-31

**Authors:** Rachid Lotfi, Hind Bel Amgharia, Sami Ennaciri, Mourad Chikhaoui, Abdeslam El kardoudi, Fatiha Chigr

**Affiliations:** 1 Department of Biology, Faculty of Sciences and Techniques, University of Sultan Moulay Slimane, Beni Mellal, Morocco; 2 Department of Biology, Faculty of Sciences Ben M’Sik, University Hassan II, Casablanca, Morocco

**Keywords:** Multiple sclerosis, pathological fatigue, physical, cognitive, psychosocial abilities

## Abstract

**Background:**

Multiple sclerosis (MS), fatigue is among the leading clinical symptoms. It is one of the most disabling symptoms for most MS people.

**Objectives:**

This research measures the prevalence of fatigue and its impact on the physical, cognitive, and psychosocial abilities of individuals with MS in Morocco.

**Method:**

This cross-sectional and descriptive study included 152 participants. Data were collected via an online survey using the snowball method, incorporating sociodemographic characteristics and the Arabic version of the Modified Fatigue Impact Scale.

**Results:**

According to the results, 89.4% of respondents experienced pathological fatigue, with an average score of 65.52 (± 18.00). There was a significant relationship between pathological fatigue, age (*p* = 0.0324), and clinical phenotype (*p* = 0.041). Fatigue negatively impacted participants’ physical, cognitive, and psychological capacities, with average scores of 70.38 (± 8.15), 62.28 (± 10.23), and 73.87 (± 1.86), respectively.

**Conclusion:**

The results of our study confirmed a high prevalence of fatigue among people with MS in Morocco. Sustained research in this field remains crucial, as it enables the formulation of strategies aimed at enhancing the quality of life for those impacted by MS.

**Contribution:**

This study is the first in Morocco to examine fatigue prevalence in people with MS and its impact on their physical, cognitive, and psychosocial abilities. High fatigue levels hinder the integration of people with MS into professional and student life. The findings emphasize the need for effective symptom and fatigue management.

## Introduction

Multiple sclerosis (MS) is a chronic autoimmune and neurodegenerative disease affecting the central nervous system (CNS). In MS, the immune system mistakenly attacks the myelin sheath, leading to inflammation, demyelination and ultimately damage to the nerve fibres themselves. This disruption in the transmission of nerve impulses can cause a wide range of symptoms, including fatigue (Lassmann 2018). Fatigue is among the main clinical symptoms of MS (Chaudhuri & Behan 2004). It affects 69% – 90% of people with MS (Giovannoni et al. 2001; Lotfi, El kardoudi & Chigr 2024; Murray 1985; Rooney et al. 2019). It is known as one of the most incapacitating symptoms of MS (Lobentanz et al. 2004). Fatigue is a subjective lack of psychoemotional or physical energy to realise daily and desired activities (Guidelines 1998). It is a reversible impairment of motor and cognitive functions accompanied by decreased motivation and a need to rest. It could occur naturally or as a result of mental or physical activity, an infection or food ingestion (Mills & Young 2008).

This symptom is multidimensional, complex and multi-causal, involving central and peripheral fatigue mechanisms (Langeskov-Christensen et al. 2017). Primary fatigue in MS is often attributed to the disease process itself, including inflammation, demyelination and axonal loss in the central nervous system (Finlayson, Preissner & Cho 2012; Kos et al. 2008; Tur 2016). Fatigue is MS’s principal risk factor affecting the quality of life (QoL) (Ciampi et al. 2018). Thus, there is a significant dependency between fatigue and the decline of QoL in MS (Barin et al. 2018; Dymecka & Bidzan 2018; Klevan et al. 2014; Leonavicius 2016; Schmidt & Jöstingmeyer 2019; Shahrbanian et al. 2015). In this regard, a survey conducted among 217 people with MS to assess the association between fatigue, sleep quality and quality of life revealed that 56.4% of respondents experienced substantial MS-related fatigue, leading to a detrimental impact on their quality of life (Tabrizi & Radfar 2015). Furthermore, MS-related fatigue adversely affects the psychological, physical, social and cognitive dimensions of people with MS (Collebrusco, Tocco & Bellanti 2018; Debouverie et al. 2007; Fernández-Muñoz et al. 2015; Göksel Karatepe et al. 2011; Grosset-Janin 2014; Moore et al. 2022; Tabrizi & Radfar 2015). Thus, fatigue can have a profound impact on various aspects of daily life, including work, social activities and personal relationships. It may limit a person’s ability to engage in physical activities, concentrate on tasks or maintain employment. This symptom can also exacerbate feelings of frustration, stress and depression in individuals with MS. It is important for healthcare providers to address the emotional and psychological impact of fatigue and provide appropriate support and resources for coping.

Furthermore, studies have revealed a relationship between fatigue and certain specific sociodemographic factors, including gender (Laabidi et al. 2019), age, education (Lerdal, Celius & Moum 2003) and occupation (Gerhard et al. 2020).

Overall, fatigue is a complex and challenging symptom of MS that requires individualised management strategies tailored to the specific needs and circumstances of each person with MS. Our study on fatigue associated with MS can contribute to advancing scientific knowledge, improving care for people with MS and raising awareness among people with MS, healthcare professionals and decision-makers to address this symptom. In addition, our investigation also helps to inform treatment decisions and better manage and alleviate the impact of fatigue in people living with MS on different aspects: physical, psychological, social and cognitive. Our study may assist in identifying potential risk factors or predictors associated with the onset or exacerbation of fatigue symptoms. This information can help healthcare professionals identify those most at risk of fatigue and implement preventative measures or early interventions. Thus, this research attempts to measure fatigue prevalence and its impact on the physical, cognitive and psychosocial abilities of individuals suffering from MS in Morocco.

## Research methods and design

### Study design

We conducted a cross-sectional and descriptive study with a sample of 152 individuals who were definitively diagnosed with MS according to current criteria. The patients included in the study come from four regions of Morocco.

### Setting

We conducted this study among patients with multiple sclerosis from the four Moroccan regions of Fez-Meknes, Marrakech-Safi, Casablanca-Settat, and Beni Mellal-Khenifra. The choice of these four regions was due to the collaboration of multiple sclerosis associations in data collection.

### Study population and sampling strategy

The study involved a sample of patients diagnosed with MS recruited from the four Moroccan regions. Inclusion criteria included a confirmed diagnosis of MS according to McDonald’s criteria, the age of the patients (18–65 years), and the ability to consent to participate in the study.

In Morocco, people with MS constitute a hard-to-reach population. For this reason, we adopted a simple, practical, and inexpensive convenience sampling method called respondent sampling, or’snowball’ sampling. This method involves a form of referral sampling, where participants are recruited through referrals from initial participants. In this case, the presidents of MS associations acted as the initial participants who recruited their members, who in turn recruited others, creating a chain or ‘snowball’ effect. We included patients with progressive MS and relapsing-remitting MS (RRMS) in our study. People whose diagnosis was not confirmed according to current criteria and those with paediatric MS were excluded from our investigation.

### Participants and recruitment

In Morocco, people with MS constitute a hard-to-reach population. For this reason, we adopted a simple, practical and inexpensive convenience sampling method called respondent sampling or ‘snowball’. This method involves a form of referral sampling, where participants are recruited through referrals from initial participants. In this case, the presidents of MS associations act as the initial participants who recruit their members, who in turn may recruit others, creating a chain or ‘snowball’ effect. We included patients with progressive MS and relapsing-remitting MS (RRMS) in our study. People whose diagnosis was not confirmed according to current criteria and those with paediatric MS were excluded from our study.

The 2017 McDonald Criteria, which include magnetic resonance imaging (MRI), clinical symptoms and laboratory findings, were used to determine the MS diagnosis (McGinley, Goldschmidt & Rae-Grant 2021).

### Data collection

We collected data via an online survey among people with MS. We used, also a platform specialised in remote scientific surveys called «Sphinxdeclic, v.4.29» for data collection. We shared the survey link in WhatsApp groups of MS associations’ members.

Integrating the online survey platform with the WhatsApp groups was an approach to enhance the validity of responses and improve participant engagement. It should also be noted that these WhatsApp groups only contain people with MS. For patients with serious physical disabilities, we contact them directly through a telephone call. Through this platform, people with MS were able to contact us personally if they did not understand the survey questions. Furthermore, we carried out a pilot survey with five people with MS to refine and finalise the questionnaire before implementing it on a larger scale. This questionnaire included sociodemographic data (age, gender, career, marital status, level of formal education, health insurance and province of residence), as well as the widely used and reliable Patient Determined Disease Steps Scale (PDDS). The PDDS is a scale of a patient-reported outcome of disability in MS and has nine ordinal levels ranging between 0 (normal) and 8 (bedridden), which assesses the degree of disability. Our questionnaire also included the Arabic adaptation of the Modified Fatigue Impact Scale (MFIS) (Khalil et al. 2019). The MFIS was adapted from the Fatigue Impact Scale (FIS), developed by Firsk et al. and validated by the Kos et al. (2005). This measure includes 21 items and examines the three components of fatigue: cognitive, physical and psychosocial.

The scores of the physical, cognitive and psychosocial subscales are added to get the final MFIS score, which ranges from 0 to 84. The score obtained out of 84 is standardised out of 100. Fatigue score is pathological when greater than or equal to 45/100.

We collect data between May and December 2022.

### Data analysis

For data analysis, IBM Statistical Package for the Social Sciences (SPSS) version 20 (multinational technology company headquartered in Armonk, New York) was used to identify the relationship between sociodemographic variables characters (age, medical coverage, school level, marital status, province of residence, gender and profession) and pathological fatigue. The Fisher Student test was used to test the association between categorical and numerical variables. We displayed data as average with standard deviations (SD). We employed multivariable analysis to investigate the relationship between sociodemographic variables and fatigue. To illustrate which sociodemographic factors are strongly linked with fatigue, we use the complete multivariable model. We use odds ratios (OR), 95% confidence intervals (CI) and *p*-values to present the results. All *p*-values had a 5% significance threshold and were two sided.

### Ethical considerations

Ethical clearance to conduct this study was obtained from the Sultan Moulay Slimane University, Biological Engineering Laboratory (No. FST/LGB/2016/28-FEB./2017-DEC.2019).

We received written informed consent from each participant for the questionnaire after describing the purposes of the research, the value of their input and their right to decline participation. The data have been anonymised and are not linked to any specific individuals.

## Results

According to the results, 75.7% of participants were women, with a mean age of 32.59 (SD ± 9.31). Additionally, the findings indicated that the survey population had an employment rate of 42.8%, compared to a national average of 39.7% for Moroccans (HCP 2021). More than 53.0% did not have any public or private medical insurance. The majority of participants (78.2%) had a university degree in terms of education (Table 1). The PDDS’s median score was 4.0, which represents a moderate level of disability. The findings indicated that the main symptoms presented by participants were fatigue (86%), cognitive complaints (70%), spasticity (62%), urinary and digestive disorders (54%), visual impairments (52%), pain (52%), mobility complaints (48%), sensory disorder (44%) and 20% present tremor (Table 1).

**TABLE 1:** Sociodemographic and clinical characteristics (*N* = 152).

Variable	*n*	%	Mean	± SD
**Gender**				
Men	37	24.3	-	-
Women	115	75.7	-	-
**Age**	-	-	32.59	±9.31
**Educational level**				
Primary	3	2	-	-
Lower and upper Secondary	24	15.8	-	-
University	119	78.2	-	-
Unschooled	6	3.9	-	-
**Marital status**				
Married	76	50	-	-
Single	67	44.1	-	-
Divorced	9	5.9	-	-
**Children**				
Yes	61	40.2	-	-
No	91	59.2	-	-
**Professional activity**				
Yes	65	42.8	-	-
No	87	57.2	-	-
**Clinical phenotype**				
PPMS	31	20.4	-	-
RRMS	121	79.6	-	-
**Patient-determined disease steps (PDDS), Median [P25, P75]**	4.0 [1.00,8.0]	-	-	-
**Symptoms**				
Fatigue	131	86	-	-
Cognition issue	106	70	-	-
Spasticity	94	62	-	-
Bowel/bladder disorder	82	54	-	-
Pain	79	52	-	-
Visual impairments	79	52	-	-
Mobility disorder	73	48	-	-
Sensory disorder	67	44	-	-
Tremor	30	20	-	-

*n*, number; RRMS, Relapsing-remitting; MS, Multiple Sclerosis; PPMS, Primary Progressive Multiple Sclerosis; s.d., Standard deviation.

With a mean score of 65.52 (SD± 18.00), the results showed that most participants (89.4%) experience pathological fatigue. In addition, the findings showed a significant relation between pathological fatigue and age (*p* = 0.0324): older participants were more exposed to pathological fatigue) and clinical phenotype (*p* = 0.041): there was an association between the presence of fatigue and progressive MS form. For the relationship between fatigue and other sociodemographic variables, the results showed no statistically significant relationship. However, in general, we found that women (OR = 2.03), married (OR = 3.22), study participants who had children (OR = 3.38), and study participants without jobs (OR = 1.78) were more exposed to pathological fatigue (Table 2).

**TABLE 2:** Fatigue description according to sociodemographic characteristics (*N* = 152).

Variable	MFIS ≤ 45 (*n*)	MFIS ≥ 45 (*n*)	Odd-ratio	*p*
Total scale	16	136	-	≤ 0.0001
**Gender (*n*)**				
Men	6	31	2.03	0.333
Women	10	105	-	-
Mean age (SD)	27 (5.73)	33.26 (9.45)	-	0.0324
**Marital status (*n*)**				
Single	10	66	3,23	0.1195
Married	3	64	-	-
**Educational level**				
Primary	0	3	-	0.851
Secondary	3	21	-	-
University	13	106	-	-
Unschooled	0	6	-	-
**Children (*n*)**				
No	13	78	3.22	0.1149
Yes	3	58	-	-
**Medical coverage (public and private) (*n*)**				
No	0	15	-	0.247
Yes	16	121	-	-
**Profession activity (*n*)**				
Yes	9	57	1.78	0.4186
No	7	79	-	-
**Clinical phenotype**				
RRMS	6	25	2.66	0.041
PPMS	10	111	-	-

Note: MFIS ≥ 45/100 = pathological fatigue; MFIS ≤ 45/100 = non-pathological fatigue; Significance tests by odds ratio, Fisher and Student test.

*n*, number; MFIS, Modified Fatigue Impact Scale; RRMS, Relapsing-remitting Multiple Sclerosis; PPMS, Primary Progressive Multiple Sclerosis.

With a mean score of 70.38 (SD ± 8.15), pathological fatigue affecting physical capacity affected most participants (84.2%). In addition, 75% of study participants suffered from cognitive fatigue, with a mean score of 62.28 (SD ± 10.23), and 88.2% experienced psychological fatigue, with a mean score of 73.91 (SD ± 1.86) (Table 2).

According to the results, physical, psychosocial and cognitive ability were negatively correlated with fatigue (*p* ≤ 0.0001). The analysis showed no statistically significant association between physical, psychosocial and cognitive ability and sociodemographic characteristics such as gender, age, marital status and job. However, the cognitive and psychosocial ability of women (OR = 3.15, OR = 2.45) and inactive participants (OR = 1.45, OR = 3.00) were more affected by pathological fatigue (Table 3).

**TABLE 3:** Fatigue description according to the cognitive, physical and psychosocial dimensions (*N* = 152).

Dimensions of fatigue	MFIS ≤ 45 (*n*)	MFIS ≥ 45 (*n*)	Mean scale	OR	*p*
**Total scale**	16	136	65.52	-	≤ 0.0001
**Physical ability**	24	128	70.38	-	≤ 0.0001
Men	6	31	-	1.04	0.9604
Women	18	97	-	-	-
Mean age (SD)	29.56 (6.99)	33.15 (9.60)	-	-	0.925
Married	9	58	-	0.83	0.7445
Single	12	64	-	-	-
Profession activity (Yes)	13	52	-	1.9	0.2488
Profession activity (No)	10	76	-	-	-
**Cognitive ability (*n*)**	38	114	62.28		≤ 0.0001
Men	16	21	-	3.15	0.0145
Women	15	62	-	-	-
Mean age	33.27 (±9.74)	32.36 (±9.21)	-	-	0.329
Married	15	52	-	0.76	0.5549
Single	21	55	-	-	-
Profession activity Yes	19	46	-	1.45	0.4131
Profession activity (No)	19	67	-	-	-
**Psychosocial ability**	18	134	73.87	-	≤ 0.0001
Men	7	30	-	2.45	0.1413
Women	10	105	-	-	-
Mean age (SD)	32.67 (4.89)	32.58 (9.76)	-	-	0.925
Married	6	61	-	0.73	0.6453
Single	9	67	-	-	-
Profession activity (Yes)	12	54	-	3.00	0.0798
Profession activity (No)	6	81	-	-	-

Note: MFIS ≥ 45/100 = pathological fatigue; MFIS ≤ 45/100 = non-pathological fatigue; Significance tests by odds ratio, Fisher and Student test.

*n*, number; MFIS, Modified Fatigue Impact Scale.

## Discussion

Our study showed a significant correlation between MS and fatigue. 89.4% of respondents had pathological fatigue, with a mean score of 65.52 (SD ± 18.00). Our results indicated that the prevalence of fatigue in Morocco is considerably higher than that reported in other studies conducted in Africa and Europe. This may be due to difficulties in accessing treatment. In Morocco, between 70.4% and 89.6% of people with MS do not have access to disease-modifying therapy (Lotfi, Chigr & Najimi 2022a; Lotfi et al. 2022b), given that the average cost of treatment is almost $1200 (Lotfi et al. 2024). For instance, fatigue was present in 65.0% of people in Tunisia (Gharsallah et al. 2023), 66.2% in Algeria (Khellaf et al. 2017) and 56.2% in Iran (Tabrizi & Radfar 2015). An earlier study in France found an average fatigue score of 57.7 (Debouverie et al. 2009), while another study in the United Kingdom (UK) found a fatigue rate of 55% (Moore et al. 2022).

Regarding the relationship between sociodemographic characteristics and the level of fatigue, the results indicated that women were more affected than men. This finding is consistent with the study by Laabili and his colleagues (Laabidi et al. 2019), which revealed a female predominance in fatigue suffering. However, another study showed that severe fatigue is more common in men (Hadjimichael, Vollmer & Oleen-Burkey 2008). These contradictory findings demonstrate the intricacy of the relationship, which is impacted by a wide range of factors, including biological, psychological and socio-cultural variables between gender and fatigue. Differences in study populations, methodologies, context of the research or definitions of fatigue may contribute to these discrepancies. Further research is needed to better understand the underlying mechanisms and determinants of differences in fatigue between men and women in different populations and conditions.

In addition, our survey revealed a positive association between fatigue and age. This outcome is consistent with several studies that have established the correlation between fatigue in MS and age: study participants with pathological fatigue tended to be older (Hadjimichael et al. 2008; Khellaf et al. 2017; Lerdal et al. 2003; Mahmoud et al. 2019; Marchesi et al. 2020; Riccitelli et al. 2021). In the same context, the finding showed that progressive MS form was statistically correlated to fatigue. This finding is also revealed by Mahmoud and his colleagues in Tunisia (Mahmoud et al. 2019). Several factors could influence the relationship between age and fatigue severity in MS. Fatigue severity may be influenced by the duration of MS (Razazian et al. 2014); older individuals with longer disease duration might experience more severe fatigue compared to younger individuals who are earlier in their disease course. With advancing age and disease duration, individuals with MS may accumulate more physical disability, which can contribute to increased fatigue. Furthermore, older individuals with MS may be more likely to have comorbid health conditions, such as cardiovascular disease or arthritis, which can exacerbate fatigue symptoms (Finlayson, Preissner & Cho 2013). Age-related cognitive changes or cognitive impairment associated with MS may impact how individuals perceive and cope with fatigue (Cameron et al. 2014). Additionally, side effects of medications used to manage MS symptoms may contribute to fatigue (Ayache & Chalah 2017). Finally, age-related life changes, such as retirement or changes in social support networks, may influence fatigue levels.

Furthermore, our findings showed that married study participants were more affected by pathological fatigue. This result concurs with the results of a study conducted by Ustunova and Ünsar (2021), who reported that more than 91.1% of married study participants suffer from fatigue. In addition, our result indicated that study participants without professional activity suffer more from pathological fatigue. Several studies show that individuals with pathological fatigue are usually unemployed (Gerhard et al. 2020; Hadjimichael et al. 2008; Ustunova & Ünsar 2021).

The findings demonstrated that the physical state of most study participants has unfavourable effects from fatigue. Thus, MS-related fatigue adversely affects physical capacity (Moore et al. 2022). Collebrusco et al., in their study, revealed a negative fatigue effect on the physical ability of study participants, with an average score of 69.44 (Collebrusco et al. 2018). Another study showed that people with MS suffer from several problems because of the effects of fatigue on their physical condition (Göksel Karatepe et al. 2011). Furthermore, study participants with lower physical activity have high intense fatigue levels (Rzepka et al. 2020).

The analysis showed no statistically significant association between physical, psychosocial and cognitive ability and sociodemographic characteristics such as gender, age, marital status and job. However, these abilities were significantly affected by pathological fatigue in women and non-active study participants. This finding was consistent with the investigation of Debouverie and his colleagues, whose mean cognitive function score was 46.8 (Debouverie et al. 2007). Furthermore, another study showed that fatigued study participants most often have a cognitive complaint compared to those who do not (Grosset-Janin 2014). In addition, the results also showed a significant influence of fatigue on the cognitive dimension of study participants. In this regard, some characteristics, such as gender and professional activity, can promote cognitive fatigue.

Our results reported that the impact of fatigue was very significant on the psychosocial state of study participants. This finding was consistent with the one published by Tabrizi and Radfar, who reported that the mean social dimension score is 61.76 (Tabrizi & Radfar 2015). Moreover, fatigue stands as one of the primary factors prompting people with MS to consider early retirement from their employment (Schiavolin et al. 2013). Göksel and his colleagues also indicated that fatigue influences the emotional state of persons with MS (Göksel Karatepe et al. 2011). In the same direction, the present study reveals that specific factors such as gender and professional activity can promote psychosocial fatigue.

From the above, it appears that fatigue is one of the most debilitating symptoms experienced by people with MS. Consequences of fatigue can have a significant impact on their physical, cognitive, emotional and social functioning. These consequences require personalised approaches. Strategies adopted in fatigue management in people with MS include medication management (Marchesi et al. 2022), regular exercise (Razazian et al. 2020), energy conservation techniques (Harrison et al. 2021), sleep hygiene (Akbarfahimi et al. 2020), stress management (Shohani et al. 2020), nutrition and hydration (Azzolino et al. 2020), pacing activities (Abonie & Hettinga 2021), cognitive behavioural therapy (Shareh & Robati 2020), support groups (Mikula et al. 2020) and regular follow-up (Kratz et al. 2020). Furthermore, various studies have explored the potential benefits of traditional and complementary medicine in managing symptoms associated with MS, including fatigue (Hasanpour Dehkordi 2016; Irish et al. 2017; Lotfi et al. 2024; Siev-Ner et al. 2003).

### Study critique

The importance of our study lies in its pioneering efforts to investigate the prevalence of fatigue in people with MS in Morocco and to assess its impact on various aspects of their lives, including physical, cognitive and psychosocial. By highlighting the challenges encountered by people with MS in Morocco, particularly in terms of integration into professional and student life, our study highlights the importance of addressing symptom management, particularly fatigue. Additionally, this study fills an important gap in the literature by providing valuable information on the prevalence and impact of fatigue in people with MS in the country. It lays the foundations for future studies and clinical interventions adapted to the needs of Moroccan people with MS. Through this study, we describe the frequency and effects of fatigue in people with MS, which helps healthcare stakeholders understand the difficulties faced by people with MS in Morocco. This could lead to more accurate identification of fatigue as a disabling symptom and the creation of targeted strategies to deal with it. We also highlighted the negative impact of fatigue on the ability of people with MS to integrate into their professional and academic lives. Employers, schools, and legislators need to know this information to put support and accommodations in place that help people with MS participate in many facets of society. Effective management of fatigue can significantly improve the quality of life and functional abilities of people with MS.

Despite the work’s advantages, there are certain limitations. including the sampling method, which could hurt the generalisation of the results, and the difficulties in reaching people with this disease in direct contact, which required us to conduct online surveys for data collection.

## Conclusion

The results of our study confirmed a high prevalence of fatigue among people with MS in Morocco. Additionally, our study highlighted the multifaceted impact of fatigue on people with MS, affecting not only physical functioning but also cognitive and psychosocial well-being. Our results also identified associations between pathological fatigue and specific MS characteristics, such as age and clinical phenotypes, providing valuable data on potential risk factors or underlying mechanisms contributing to fatigue associated with MS. Ongoing research is needed to better understand the underlying mechanisms of fatigue in MS and develop more effective interventions to alleviate its burden on those living with the condition.
